# Improving the Deployment of WSNs by Localized Detection of Covered Redundant Nodes in Industry 4.0 Applications

**DOI:** 10.3390/s22030942

**Published:** 2022-01-26

**Authors:** Marwa Hussein Hamad Aljubori, Vahid Khalilpour Akram, Moharram Challenger

**Affiliations:** 1 International Computer Institute, Ege University, Izmir 3100, Turkey; marwarija88@gmail.com (M.H.H.A.); vahid.akram@ege.edu.tr (V.K.A.); 2 Department of Computer Science, University of Antwerp, 2020 Antwerp, Belgium; 3 Flanders Make Strategic Research Center, 3001 Leuven, Belgium

**Keywords:** wireless sensor networks, coverage redundancy, Industry 4.0, redundant nodes

## Abstract

Wireless sensor networks can be used as cost-effective monitoring and automation platforms in smart manufacturing and Industry 4.0. Maximizing the covered area and increasing the network lifetime are two challenging tasks in wireless sensor networks. A feasible solution for maximizing the coverage area and network lifetime is detecting and relocating the covered redundant nodes. A covered redundant node is a node whose covered area is also covered by the other active nodes in the network. After identifying the covered redundant nodes, putting them in sleep mode can increase the network lifetime. In addition, moving the detected redundant nodes to the uncovered locations can improve the overall covered area by the sensor nodes. However, finding the redundant nodes is an NP-complete problem. In this paper, we propose a localized distributed algorithm for identifying the redundant nodes based on the 2-hop local neighborhood information of the nodes. The proposed algorithm uses the existing connections between the neighbors of each sensor node to decide the redundancy of the node. The algorithm is localized and does not need the entire topology of the network or the coordinates of the nodes.

## 1. Introduction

Industry 4.0 and smart manufacturing can improve the efficiency and productivity of the factories by automating the manufacturing operations, monitoring the events, and making real-time intelligent decisions based on the received feedback from the devices. Smart manufacturing can help to improve the quality of products, reduce pollution and costs, and improve workplace conditions. Wireless sensor networks (WSNs) are among the most promising platforms for monitoring and automation in smart manufacturing and industry 4.0. A WSN may consist of thousands of low-cost small devices generally called sensor motes. The sensor motes can sense different events and values, such as temperature, pollution level, light, pressure, and environmental movements, and send these data to a processing center. Old sensory units were expensive and had limited sensing, communicating, and processing capabilities, but the current smart sensor motes can sense different values, process and store data, or forward the collected data over radio messages to the other nodes. Recent sensor nodes are usually equipped with one or more sensing modules, a wireless communication module, memory, and a low-power processor.

**Figure 1:**
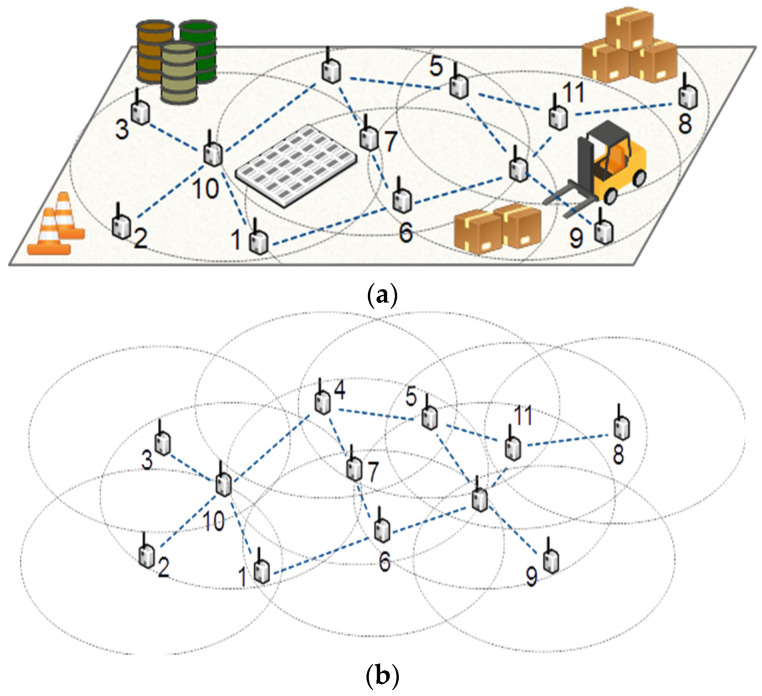
(**a**) A sample WSN, (**b**) covered area by each node.

WSNs can be used in many applications, such as smart manufacturing, agriculture, target tracking, health care, and industrial applications [1,2]. Generally, in WSNs, the sensing and radio communication range of sensor motes are limited, and many nodes should be distributed in the environment to monitor the desired events or properties. Using different medium access protocols [3], the motes may avoid packet collisions and securely send their collected data to a base station. Figure 1a shows a sample WSN where the dashed big circles are the coverage range and the edges between nodes are the communication channels. Because of the importance of real-time and complete monitoring in most real-life applications, we need to ensure that all points in the target environment or area of interest are within the sensing coverage of at least one sensor node. Covering the entire area of the target region is generally known as the full coverage problem. Because of the importance of full coverage in most applications, this problem has been the subject of many studies from different perspectives, such as node deployment, power assignment, coverage restoration, and coverage hole detection. Usually, some regions are not covered by the sensor nodes due to different reasons, such as random distribution, sensor destruction, or battery drain. These uncovered regions are called coverage holes. A feasible solution to reduce the coverage holes with limited resources is detecting and relocating the redundant nodes, which has a negligible effect on the overall coverage area. This paper proposes a method to detect the redundant nodes whose covered area is fully covered by the other nodes, so they do not affect the overall coverage area. For example, in Figure 1b, the covered area of node 7 has been entirely covered by the other nodes. Therefore, in case of node failure and coverage loss, we may move node 7 to the location of the damaged node to restore the coverage. For example, in Figure 1b, if node 3 stops working for any reason, we lose the coverage of a relatively wide area. In this case, moving node 7 to the location of node 3 may eliminate or minimize the coverage loss. We use the 2-hop local subgraph of nodes to estimate the other nodes’ covered area.

After detecting the redundant covered nodes, we may put them in sleep mode to save their energy for future tasks. In the case of failure in critical nodes, we may move the nearest redundant node to the location of the damaged node to restore the coverage. In this way, we may use the available resources more efficiently and maximize the coverage range and lifetime of WSNs as much as possible. The remaining parts of this paper have been organized as follows: Section 2 provides a survey about related works, Section 3 includes the theoretical foundation of the proposed method, the proposed covered nodes detection algorithm is presented in Section 4, Section 5 includes the performance evaluation, and finally, Section 6 draws the conclusion.

## 2. Related Works

**Figure 2:**
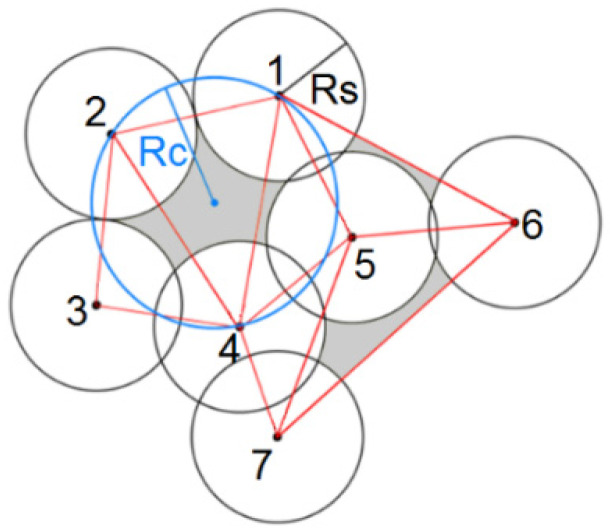
Delaunay triangles with the empty circle.

In wireless sensor networks, detecting the uncovered areas, finding the redundant nodes, and maximizing the covered area are important and challenging problems. The coverage holes detection approaches can be divided into geometrical, topological, and statistical methods. Geometrical approaches use the two-dimensional coordinate information of nodes and various geometrical tools to identify the uncovered area. Some studies use the Voronoi diagrams and Delaunay triangulation to identify the uncovered areas. Through the Delaunay triangulation method, a Delaunay triangle is created by linking the three centers of the neighbor sensor nodes. An empty circle of a Delaunay triangle is a wraparound circle that does not contain any vertex of other Delaunay triangles. For example, Figure 2 shows a sample WSN where Rs is the radius of the sensing range of sensor nodes and Rc is the radius of the resulting circle from nodes 1, 2, and 4. If Rc is bigger than Rs, we have some uncovered area inside the established circle [4]. The proposed approach in [5] has used Delaunay triangulation to locate the coverage holes under random deployment of nodes and proposed an approach for estimating the nodes that are located on the boundary of coverage holes by the Voronoi diagram. The proposed method in [6] estimates the coverage holes by establishing spanning trees on the network. This tree-based method creates small empty circles within each Delaunay triangle to accurately determine the size of the holes. The authors of [7] have proposed an algorithm based on Delaunay triangulation that adds a virtual edge between nodes to approximate the coverage holes in a deployed WSN. Qiu et al. have proposed another distributed algorithm that is based on Delaunay triangulation. The algorithm is coordinate-free and can detect the uncovered area when the accurate location coordinates of nodes are not available [8]. Ma et al. have proposed another distributed method that benefits from computational geometry for detecting the uncovered areas as a post-deployment process [9].

Topological approaches use topological properties of the networks, such as connectivity, centrality, and degree of nodes, to identify the coverage holes and boundary nodes. Bi et al. have proposed two neighborhood-based algorithms for coverage hole detecting in WSN without using the location information of the nodes [10,11]. Another topological hole detection algorithm has been proposed in [12] that identifies the nodes near the boundary of the sensor field and coverage holes. Beghdad et al. have used the topological methods and existing connections between the nodes to detect the borders of uncovered areas in the network [13]. Their proposed method uses a connected independent set algorithm to detect the coverage holes in WSNs. The statistical approaches for coverage hole detection do not require topological or location information but require dense sensor nodes deployed in the target environment. For example, the proposed method in [14] uses a graph clustering technique to divide the nodes in the network into small groups that reveal the connections and coverage holes. After identifying the groups, the nodes in the borders of each group are detected using betweenness centrality scores, and clusters are grouped according to their closeness. The nodes located at the border area of different groups will not be around any coverage hole. Therefore, the algorithm allows the detection of nodes located at the boundary of clusters. Amgoth et al. have proposed another distributed algorithm for detecting and restoring the uncovered areas in the wireless sensor networks [15]. The nodes create local clusters in their proposed algorithm and detect the coverage hole based on local cluster information. Then, the nodes close to a coverage hole decide independently to restore it by increasing their sensing range.

Finding the covered redundant nodes is another crucial problem that can help to increase the network lifetime and coverage area of WSNs. In dense sensor networks, activating only a subset of sensors at a time can increase the overall network lifetime. However, determining all redundant nodes is an NP-complete problem [16]. Hence, different approaches have been proposed for estimating a subset of covered redundant nodes using different heuristics. For example, the proposed method in [16] finds the spatially correlated nodes set to determine the subset of active nodes at a given time. As another example, the proposed method in [17] presents a distributed node scheduling mechanism to estimate the redundant nodes based on the positions of neighbor nodes. After detecting the redundant nodes, the algorithm puts them in sleep mode to increase the network lifetime. The algorithm can only detect a subset of redundant nodes and needs the exact location and the sensing radius of nodes. A probabilistic method for detecting the redundant nodes has been proposed in [18], which calculates a covering probability for each node based on the coverage and density around a node. This method needs the location information of the nodes and provides a probability model that calculates a likelihood value for each node, which estimates its redundancy status.

To reduce redundant sensing in WSNs, mathematical modelling has been proposed in [19], which uses biologically inspired techniques to minimize the overlapped sensing area. These models define an objective function for an overlapping area and use genetic and ant colony algorithms as meta-heuristics to find optimal answers for objective function. Similarly, different objectives and a lexicographic evolutionary algorithm have been proposed in [20] to minimize redundant coverage. The proposed approach in [21] detects the redundant sensed data based on the pattern generation approach and reduces source nodes’ sensing range. The proposed redundancy-aware topology control protocol in [22] finds the redundant sensor nodes from connectivity. The proposed protocol identifies the backbone nodes and turns of the other nodes to increase the network lifetime. The proposed algorithm in [23] uses ant colony optimization to reduce the sensing cost while maintaining the network’s coverage and connectivity. A sleep/awake schema based on redundant data and duty cycling has been proposed in [24] that tries to reduce the overall energy consumption of nodes and increase the network lifetime. Generally, the proposed methods in [20,21,22,23,24] focus on discovering the redundant data or redundant covered area.

In contrast, we focus on detecting the redundant covered nodes in this paper. Another sleep/awake scheduling algorithm has been proposed in [25] that tries to identify a set of nodes that cover the target area and put the remaining nodes in sleep mode until the failure of an active node. The proposed algorithm is based on an optimization method and needs the entire topology graph. Our proposed method is distributed and uses local neighborhood information to identify the redundant nodes. The proposed method in this paper does not need the location information of the nodes and only uses the local neighborhood information. Unlike the probabilistic models, the proposed method’s detected nodes are assuredly redundant in the network. Improving the coverage and connectivity robustness between the nodes are two vital requirements for using WSNs in Industry 4.0 [26,27]. Therefore, detecting the redundant covered nodes may help to improve the coverage performance of WSNs and increase their usability in Industry 4.0 applications.

## 3. Theoretical Foundation

Generally, a WSN is modelled as a graph G (V, E), where V is the set of nodes and E is the set of communication links between the nodes. We refer to the 1-hop neighbors of node v with $\Gamma_{v}$ and the 2-hop subgraph of node v with $G_{v}$. In addition, we show the number of nodes and maximum degree of nodes with n and Δ, respectively. At the end of the proposed algorithm, each node keeps its redundancy state in the status variable, where the value of this variable can be covered or uncovered. Table 1 presents the used notations and their meaning in this paper. 

The nodes in the radio range of each other may have a communication link and can send radio messages to each other. Besides the communication range, each node has a sensing range that determines the covered area by the nodes. The range of communication and sensing range of nodes depend entirely on the type of sensors and communication channels. In the proposed algorithm, we have the following assumption about the network:

1. Each sensor node has a unique identifier.
2. The nodes may broadcast a message to their neighbors.
3. The communication range (radius) of all sensor nodes is almost equal.
4. The sensing range (radius) of all nodes is almost equal.
5. The sensing range (radius) of nodes is equal to or larger than their communication range.

**Figure 3:**
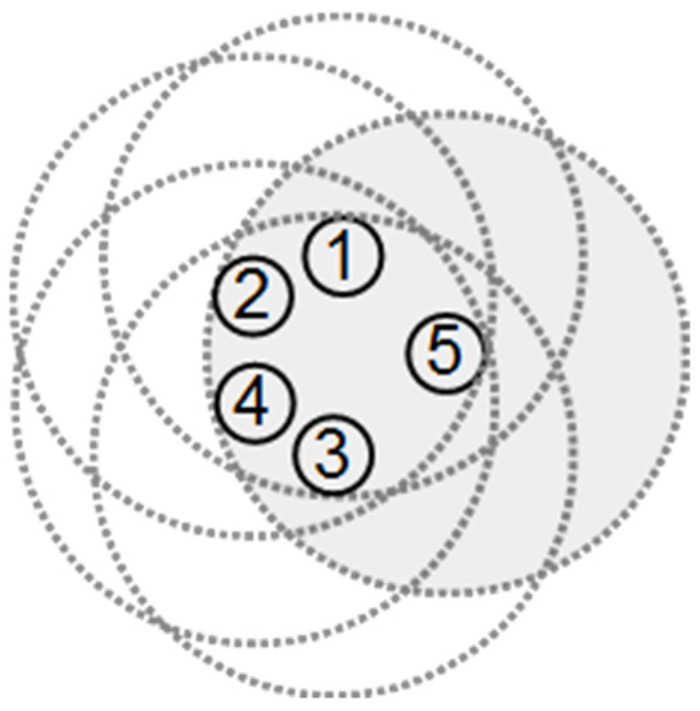
All neighbors of node 5 are in the radio range of each other (1–5 are the nodes ID).

To achieve the maximum covered area, we have to deploy the nodes as far as possible from each other. However, the communication range of the nodes limits the maximum distance between the nodes. Furthermore, in some applications, the nodes are randomly distributed in the environment, leading to many coverage holes or redundant nodes. If all nodes in $\Gamma_{v}$ connect to every other node in $\Gamma_{v}$, then removing v may create a coverage hole. For example, in Figure 3, we have $\Gamma_{5}=\left\{1,2,3,4\right\}$, and each node in $\Gamma_{5}$ has a link to all other nodes in $\Gamma_{5}$. In this network, removing node 5 can lead to a coverage hole. 

**Figure 4:**
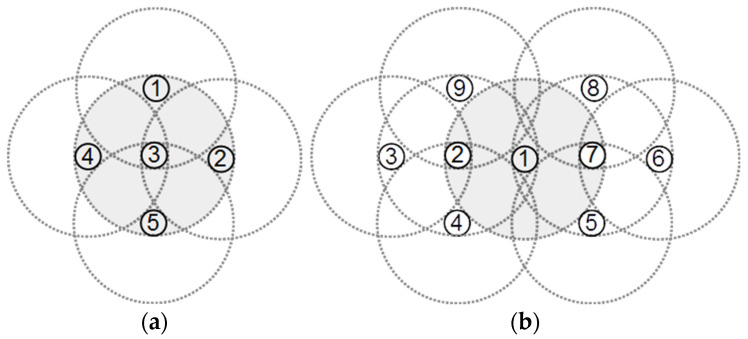
(**a**) The four neighbors of node 3 are not connected and completely cover node 3; (**b**) the two neighbors of node 1 are not connected, and their neighbors completely cover node 1 (1–7 are the nodes ID).

Generally, if most of the nodes in $\Gamma_{v}$ are connected, then the neighbors of nodes are close to each other, which means that multiple nodes cover some area around node *v*s, and some other area may only be covered by node v. If the neighbors of node v are far from each other, they may cover more area around node v. This paper proposes two rules for estimating the covered area by more than one node and finding the covered redundant nodes. The first rule is based on the number of interconnected neighbors of node v. If node v has at least four neighbors in $\Gamma_{v}$ without a direct edge between each other, then the covered area of v is completely covered by its neighbor nodes. For example, Figure 4a presents an example network where V={1,2,3,4,5} and E={(1,3),(2,3),(4,3),(5,3)}. The dashed big circles in Figure 4a show the radio and sensing range of the nodes. In this figure, node 3 has four neighbors, and none of them have a link to each other. Figure 4a shows that all area covered by node 3 has also been covered by its 1-hop neighbors. If the sensing and communication range of all nodes is equal, and node u has at least four non-adjacent neighbors, then its neighbors are far enough from each other to remain out of communication range and close enough to u to be its neighbor. This implies that each neighbor of node u covers at least a disjoint quarter of the area covered by node u, which means that all area covered by node u is covered by its non- adjacent four neighbors. Therefore, we may conclude that if a node has at least four non-connected neighbors, its covered area is completely covered by the other nodes. In this case, the node can be considered a covered redundant node and may be moved to the location of other nodes or go into sleep mode to minimize its energy consumption.

Our proposed second rule is based on the number of detected covered neighbors of each node. If node v has at least two detected covered neighbors, then v is a covered redundant node. Figure 4b presents a sample network in which nodes 2 and 7 may decide that they are redundant using rule 1. Therefore, node 1 will have two covered redundant neighbors, which means that its 2-hop neighbors are far enough from each other to cover the area of node 1. Figure 4b shows that in this case, the covered area of node 1 will be fully covered by its 1- and 2-hop neighbors. Therefore, if a node has at least two covered redundant neighbors, it may mark itself as covered redundant. After detecting the redundant covered nodes, we may put some of them into sleep mode or move them to the location of other failed nodes to increase the general coverage of the WSN. Note that some of the redundant nodes may have a critical role in keeping the connectivity between other nodes in the network. Therefore, before moving or sleeping a redundant node, we need to ensure that the network remains connected without that node. To do this, we may start a distributed critical nodes detection [28] or a distributed minimum cut detection algorithm [29] to identify the nodes whose removal divides the network to the disconnected partitions. In this way, we may keep the redundant critical nodes at their initial location and move the other redundant nodes. 

Additionally, removing redundant nodes may generate some network coverage holes. For example, in Figure 4b, if we remove nodes 2 and 7, as they are redundant by rule 1, node 1 will not be redundant anymore. Therefore, after identifying the redundant nodes, we may select a subset of redundant nodes for moving such that they are at least 2-hop from each other. In this way, removing the redundant nodes will not generate any holes in the network.

## 4. Proposed Algorithm

In the proposed algorithm, each node explores its 2-hop local subgraph and then decides whether it is a covered redundant node based on the links between its 1 and 2-hop neighbor nodes. Initially, all nodes broadcast a “Hello” message, allowing each node to identify its 1-hop neighbors. After finding the list of 1-hop neighbors, each node broadcasts an NGB message, allowing its neighbor nodes to construct their 2-hop local subgraph. After receiving NGB from all neighbors, each node detects its status (covered or not covered) using the proposed first rule. Then, the covered nodes broadcast a “Covered” message, which allows its neighbors to check the second rule.

**Figure 5:**
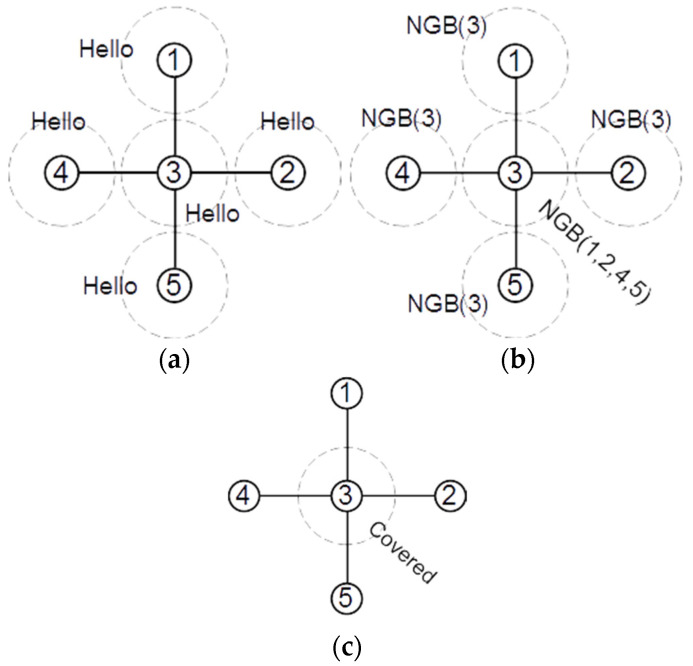
(**a**) All nodes broadcast a “Hello” message; (**b**) all nodes broadcast their neighbor list; (**c**) node 3 marks itself as a covered redundant node and broadcasts a “Covered” message (1–5 are the nodes ID).

**Figure 6:**
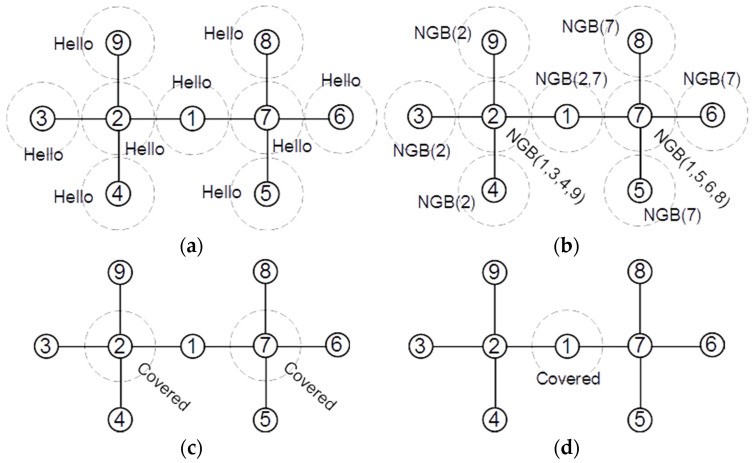
Finding nodes covered by two covered neighbor nodes. (**a**) All nodes broadcast Hello message. (**b**) All nodes broadcast neighbor list. (**c**) nodes 2 and 7 broadcast Covered message. (**d**) after receiving Covered from 2 and 7, node 1 broadcasts Covered message (1–9 are the nodes Id).

Figure 5 shows the broadcasted messages by the nodes in a sample network. Initially, each node in a network broadcasts a “Hello” message, which allows the nodes to find their 1-hop neighbor list (Figure 5a). When node v receives a “Hello” message from node u, it adds the ID of node u to its local $\Gamma_{v}$ set. For example, after receiving “Hello” messages, we will have $\Gamma_{3}=\left\{1,\;2,\;4,\;5\right\}$ and $\Gamma_{1}=\left\{3\right\},\;\Gamma_{2}=\left\{3\right\},\;\Gamma_{3}=\left\{3\right\}$ and $\Gamma_{5}=\left\{3\right\}$. After creating the 1-hop neighbor list, each node sends its neighbor set in an NGB message (Figure 5b). After receiving the NGB messages, each node constructs its 2-hop subgraph in its memory and checks the number of connections between its 1-hop neighbors. If at least four neighbors of node v have no connection to any other node in $\Gamma_{v}$, node v marks itself as a covered redundant node and broadcasts a “Covered” message (Figure 5c). Figure 6 shows the broadcast messages by the nodes in another network. In the first step, all nodes broadcast a “Hello” message and find their 1-hop neighbor list (Figure 6a). In the second step, all nodes broadcast their 1-hop neighbor list in an NGB message (Figure 6b). Then, each node that has at least four neighbors without direct connections marks itself as covered redundant and broadcasts a “Covered” message (Figure 6c). Finally, each node that receives at least two “Covered” messages from its neighbors marks itself as covered redundant and broadcasts a “Covered” message (Figure 6d).

Algorithm 1 presents the main steps of the proposed distributed method. In this algorithm, we refer to the sender node as node u and the receiver node of a message as node w. Initially, all nodes broadcast a “Hello” message (line 1). Each node that receives a “Hello” message from a neighbor inserts the sender’s ID to a local set (line 3). After receiving the first “Hello” message, each node sends its neighbor set after t time unit. This time ensures that the nodes receive all broadcast “Hello” messages (lines 4,5). After receiving an NGB message from node u, node w updates its local graph by adding u and its connected neighbors to the graph (lines 7,8). After receiving an NGB message from all neighbors, the receiver node w calls the DetectStatus procedure to detect its status (lines 9,10). The DetectStatus procedure follows our proposed rules to detect the status of each node. If the receiver node has at least four neighbors in the 1-hop neighbor set, and they have no direct link to the other three nodes, then the node sets its status as a covered node and broadcasts a “Covered” message (lines 11–14). If an uncovered node receives at least two “Covered” messages from its neighbors, it changes its status to covered and broadcasts a “Covered” message (lines 16–18). After detecting the covered redundant nodes, we may identify the critical redundant nodes, whose removal disconnects the network, and put the remaining non-neighbor redundant nodes in sleep mode to save their energy or move them to the location of the other failed nodes.
**Algorithm 1: Covered Redundant Nodes Detection**.1: All nodes broadcast a “Hello” message. 2: **Upon receiving a “Hello” message from**
u
**in**
w: 3: $\Gamma_{w}\;\leftarrow \Gamma_{w}\;\cup \left\{u\right\}$. 4: $\mathrm{If}\;|\Gamma_{w}$| = 1 then 5: ***Broadcast***
$\mathrm{NGB}\;(\Gamma_{w}$) *after t time unit* 6: **Upon receiving NGB**($\Gamma_{u}$) **from**
u
**in**
w: 7: $G.V\leftarrow G.V\cup \left\{\Gamma_{u}\right\}.$ 8: ∀s $\in \Gamma_{u}\;$: G.E←G.E∪(u,s). 9: **if** the number of received NGB($\Gamma_{w}$) = |$\Gamma_{w}$| **then** 10: **calls**
*DetectStatus(w) procedure*. 11: **Procedure DetectStatus**(w): 12: $\mathrm{if}\;\exists p=\left\{a,b,c,d\right\}\in \Gamma_{w}$
**s.t**
 ∀ (u,v)∈p :(u,v)∉ G.E 
**then** 13: ${\mathrm{status}}_{w}\leftarrow$ covered. 14: **Broadcast** “Covered.” 15: **Upon receiving “Covered” message in**
w: 16: **if** number of received “Covered” messages > 2 **and**
${\mathrm{status}}_{w}\neq$ covered then 17: ${\mathrm{status}}_{w}\leftarrow$ covered. 18: **Broadcast** “Covered.”

To evaluate the message and space complexities of the proposed algorithm, we assume that the network has n nodes, and each node has a maximum degree of Δ. In our proposed algorithm, each node sends a “Hello” and an NGB message. Since the “Hello” messages contain only the ID of the sender node, the size of these messages is $log_{2}n$ bits. Each NGB message contains the sender’s 1-hop neighbor list and can have at most Δ items. Therefore, the size of each NGB message is $\Delta \;log_{2}n$. The size of each “Covered” message is $log{\;}_{2}n$ bits, and up to n “Covered” messages can be sent in the network. Thus, the overall message complexity of the proposed algorithm is $O(n\;\Delta \;\log_{2}n)$. Each node should keep a 1-hop neighbor list and a 2-hop local sub-graph in its memory in the proposed method. The 1-hop neighbor list can contain up to Δ items. The 2-hop local sub-graph can have up to $\Delta^{2}$ nodes. In the worst case, if each node in the local sub-graph has a link to all other nodes, we will need $O\left(\Delta^{4}\right)$ memory units. Therefore, the space complexity of the proposed algorithms is $O(\Delta^{4})$.

## 5. Performance Evaluation

**Figure 7:**
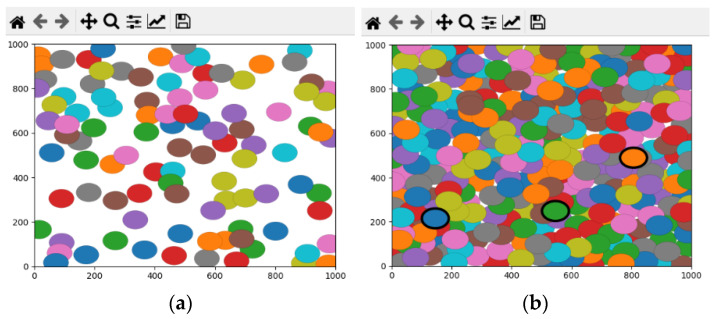

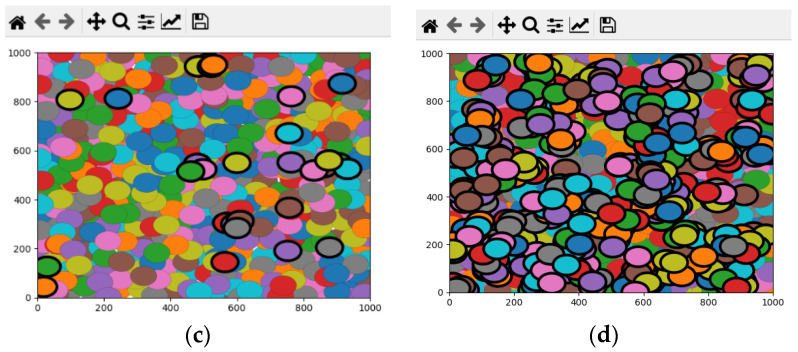
Detected covered nodes in the networks with (**a**) 100 nodes, (**b**) 300 nodes, (**c**) 500 nodes, and (**d**) 700 nodes.

We evaluated the performance of the proposed algorithm by implementing it in Python language using the Shapely and MathPlot libraries. We generated different topologies with 100 up to 800 (stepping 100) nodes deployed randomly in the network area. We set the width and height of the network area to 1000 m and selected the x and y coordinates of nodes uniformly between 0 and 1000. If the selected random position was already assigned to another node, we selected a new random position for the current node. We set the sensing range of the sensor nodes between 20 and 100 m and counted the number of detected redundant covered nodes by the algorithm. Figure 7a–d, respectively, show sample generated networks with 100, 300, 500, and 700 nodes and the detected covered nodes in these networks. In this figure, the sensing range of all nodes is 40 m, the size of the area is 1000 m × 1000 m, and the nodes with thick borderlines are the detected covered nodes.

**Figure 8:**
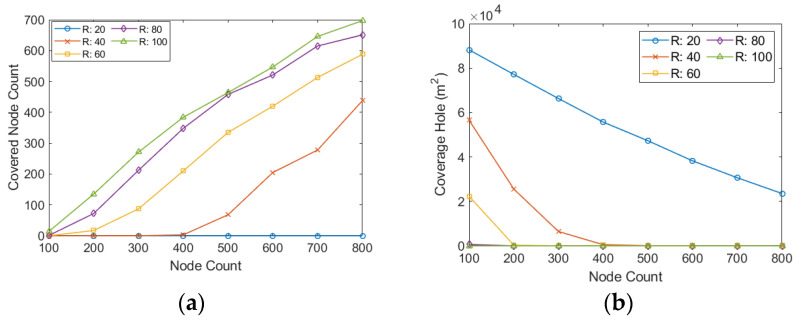
(**a**) Number of detected covered nodes against the node count; (**b**) the uncovered are of the network against the node count.

Figure 8a shows the number of detected covered nodes in a different sensing radius than the node count in the network. This figure shows that increasing the number of nodes and the sensing range increases the number of detected covered nodes. In networks with 800 nodes, almost no covered node is detected when the sensing range of nodes is 20 m. 

However, in the same network, when we increase the sensing range to 100 m, about 700 nodes are entirely covered by the other nodes. Figure 8b shows the sum of the uncovered areas in the network for different sensing ranges against the number of nodes. This figure shows that increasing the number of nodes or sensing ranges reduces the network’s uncovered area. In the networks with 100 nodes, the uncovered area of the network reaches about 87,000 m^2^ when the sensing range of nodes is 20 m. For the same sensing range, the total size of the uncovered area decreases to about 21,000 m^2^ when we add 800 nodes to the network. In the networks with more than 200 nodes and a sensing range higher than 60 m, almost the entire network area is covered by the nodes. 

**Figure 9:**
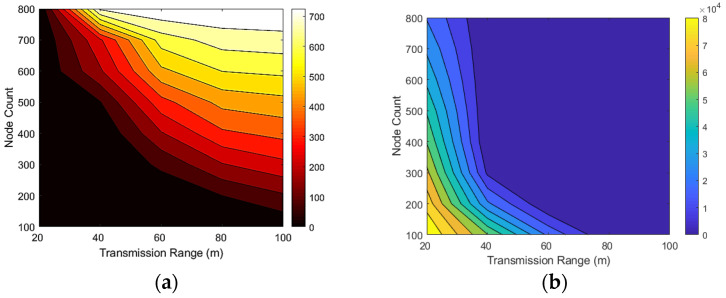
Relationship between the number of nodes and sensing ranges with (**a**) the number of detected covered nodes and (**b**) the uncovered area in the network.

Figure 9a illustrates the relation between the number of detected covered nodes and the number of nodes and sensing ranges. In this figure, dark colors are small, and light colors indicate numerous detected redundant nodes. This figure shows that increasing the number of nodes and the sensing range increases the number of covered redundant nodes. Figure 9b illustrates the relationship between the uncovered area in the network and the number of sensor nodes and sensing ranges. In this figure, the dark colors show fewer coverage holes, and the light colors show more coverage holes. Figure 9b indicates that increasing the number of sensor nodes or sensing ranges rapidly decreases the network’s uncovered area. 

**Figure 10:**
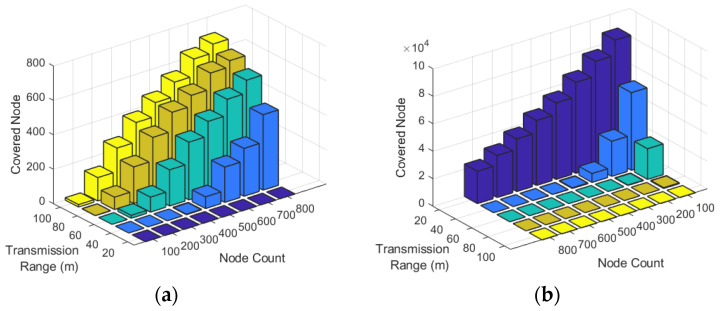
(**a**) The number of detected covered nodes and (**b**) the uncovered area in the network against the node count and sensing ranges.

Figure 10a illustrates the detected covered nodes against the number of nodes and the sensing range of nodes. This figure indicates that in the networks with more than 400 nodes and a sensing range of more than 60 m, at least 200 nodes are detected as covered redundant nodes. Finally, Figure 10b shows the total uncovered area of the network against the number of nodes and sensing range of nodes. The figure shows that when the sensing range of nodes is 20 m, more than 20,000 $m^{2}$ of the network remain uncovered. When we increase the sensing range to more than 40 m and the number of nodes to more than 300, the nodes cover most of the network.

## 6. Conclusions

This paper proposed a localized distributed algorithm to find the covered redundant nodes in the wireless sensor networks. The proposed algorithm can be used to detect the redundant covered nodes in the applications that require many sensor nodes to cover relatively large areas. Especially in harsh environments or in scenarios where regular node deployment is hard or impossible, detecting the redundant covered nodes after a random node distribution can considerably improve the covered area. A covered redundant node is a node whose neighbor sensor nodes entirely cover its covered area. Detecting the covered redundant nodes may increase the network’s coverage area by moving the detected covered nodes to the uncovered area of the network. Additionally, putting the covered redundant nodes into sleep mode may save their energy and increase the network lifetime. The proposed algorithm uses the connectivity between nodes in the 2-hop local subgraph of each node to find the fully-covered other nodes. The simulation results show that the proposed algorithm can find the most redundant nodes in the dense network. In the proposed algorithm, each sensor node sends a few short messages that reduce the total consumed energy to detect the redundant covered nodes. 

The proposed algorithm relies on the local neighborhood information; therefore, it may miss some redundant nodes. After detecting the redundant nodes, we need to identify the critical nodes that keep the network’s connectivity and select the non-critical non-neighbor redundant nodes for sleeping or relocating. As for the future works of this research, we plan to propose efficient algorithms for detecting the uncovered area of the network and optimal moving of the detected covered nodes to uncovered areas to maximize the total coverage area.

## Figures and Tables

**Table 1:** Notations and their meaning.

Symbol	Meaning
G(V,E)	Topology graph including nodes set V and edges set E.
$\Gamma_{v}$	1-hop neighbor set of node v.
Δ	Maximum node degree.
n	The number of sensor nodes in the network.
${\mathrm{status}}_{v}$	The covered or uncovered status of node v.

## Data Availability

Not applicable.
